# On the Influence of Section of the Cervical Pneumogastrics on the Action of Emetics and Cathartics

**Published:** 1870-11

**Authors:** 


On the Influence of Section of the Cervical Pneumogastrics on the Action of Emetics and Cathartics.—Dr. H. C. Wood, Jr., in the *American Journal of Medical Sciences*, details a very extensive series of experiments on the lower animals, to demonstrate the facts in regard to this subject, which he accompanies with copious comments.

Although the English and French anatomical works do not describe the pneumogastric as descending in the abdomen below the upper end of the duodenum, Dr. J. Rollman, a German, seems to have demonstrated, that the posterior, or right par vagum, is distributed to the liver, spleen, kidneys, supra-venal capsules, and the whole of the small intestines; hence, it seemed rational to believe a connection of function existed between them.

The first series of experiments described, was instituted to determine how far section of the vagus nerve interferes with vomiting. Four dogs of different sizes and species were dosed, some time after section of the nerves, with a variety of emetics in large potions, such as viridia, veratria, and veratroidia, and both internally and hypodermically, and they all died without vomiting. Three other dogs were experimented with in the same way, only that antimony was given as the emetic, with like results. Ten other experiments were made with lethal doses of veratria and with arsenic, without causing vomiting. These are sufficient to prove that emetics will not act after division of the pneumogastrics. "That the non-occurrence of emesis is caused by the arrest of gastric secretion is, I think, demonstrated by the absence of fluid in the stomach on *post-mortem* examination."

In order to determine the relation between these nerves and purgation, eighteen animals, mostly cats, were experimented with. These experiments were varied, both in the drugs used, and the manner of using, in almost every conceivable way in which purgation could possibly be produced, after the nerves were cut. Only on two, out of the eighteen, was there any purgation induced.

These experiments with the first series seem sufficient to prove that purgation is impossible after division of the cervical pneumogastrics.

Two experiments were tried in which there was purgation, very free in one and slight in the other, after division of the nerves.

Why is it that in section of these nerves in a majority of cases purgatives do not act, but in a few cases they do? The answer is evidently wrapped up in the question: How does division of the nerve cause arrest of intestinal secretion?

After section of the par vagum secretion in the stomach is arrested; the arrest of that of the intestines is doubtless due to the same cause. Three plausible explanations present themselves:

*First.* The par vagum is possibly the direct antagonist of the contracting vaso-motor nerve of the alimentary canal. If this is true, there must be contraction of the capillaries after section of the nerves preventing secretion.

*Second.* Shock.

*Third.* Accumulation of carbonic acid in the blood owing to the impeded respiration.

In order to determine whether the par vagum was a vaso-motor nerve or not, he tried other experiments, nine in number. The course pursued was to open the abdomen after section of the nerve, and apply various irritants to the intestines, both inside and outside. "A careful study of three experiments will, I think, convince any one that there is no direct connection between the pneumogastric nerve and the intestinal circulation."

In order to see how far "shock" could diminish the action of the intestinal glands, another series of experiments was made. A cat had all its legs broken, and vomited readily on administration of emetics; upon others the carotids were tied, and other means were taken to produce shock, after which emetics and carthartics were given, usually with their full operation. Dr. W. says of these last experiments, "they are not very decisive, but are, I think, sufficient to show that the intestinal effects of a division of the nerves cannot be accounted for by 'shock' alone."

To ascertain how far the arrest of secretion in the alimentary canal after division of the nerves is due to the disturbances of the respiration, to the deficient decarbonization of the blood, two series of experiments were undertaken. In the first, the action of one lung was more or less obstructed by air forced into the pleural cavity and otherwise, and then purgatives were administered. Five animals were operated upon, and in all but one, the ordinary action of the medicine was wanting; in this one there was slight purging only.

In the second series of experiments, the animals were compelled to breathe an atmosphere heavily loaded with carbonic acid gas, while they were plied with purgatives in very large doses. The trial was made with four different cats, and in not one was there any normal action of the purgatives produced.

These experiments seem to prove conclusively, that an excess of carbonic acid in the blood is capable of lessening, and when carried beyond a certain point, of wholly preventing in most cases, emesis and purgation. In doing this, the poison not only arrests muscular action, but stops likewise secretion, as is shown by the empty condition of the intestines *post mortem.*

To clear up the question as to which of these results is produced in greatest degree two cats were killed by inhaling carbonic acid, when on opening the abdomen, it was found in both cases that on irritation considerable peristaltic action was manifested.

The doctor sums up the results of his extended study as follows: "*First.* The division of the cervical pneumogastric does, in the majority of instances, but not always, absolutely arrest free gastro-intestinal secretion, emetics and cathratics being absolutely powerless to produce it.

*Second.* That this arrest is not due to any direct influence which the nerve has upon the intestine or its circulation, but is owing to two or three causes: accumulation of carbonic acid in the blood, interference with the circulation of the lungs, backing up the blood upon the portal circulation, and perhaps shock."

